# Thiazolidinediones and Cardiovascular Risk — A Question of Balance

**DOI:** 10.2174/157340309788970333

**Published:** 2009-08

**Authors:** Erland Erdmann, Bernard Charbonnel, Robert Wilcox

**Affiliations:** 1 Clinic III for Internal Medicine and Cardiology, University of Cologne, Cologne, Germany; 2 Clinique d’Endocrinologie, Hôtel Dieu, Nantes Cedex 1, France,; 3 Department of Cardiovascular Medicine, University Hospital, Nottingham, UK

## Abstract

**Background:**

Several recent meta-analyses of adverse event data from randomized controlled trials with rosiglitazone reveal a possible association between this thiazolidinedione and an increased risk of ischemic myocardial events. This has led to debate on the overall clinical benefit of glitazone therapy for type 2 diabetes. Pioglitazone, on the other hand, has the most extensive cardiovascular outcomes database of all current glucose-lowering therapies, including a large prospective randomized controlled trial designed specifically to assess cardiovascular outcomes (PROactive). The available data suggest that pioglitazone is associated with a reduction in macrovascular risk.

**Aims:**

In this review, we highlight some of the key factors that need to be considered when assessing the net clinical benefit of thiazolidinediones, focussing on both class effects and those specific to either rosiglitazone or pioglitazone.

**Results:**

For pioglitazone there appears to be no increase in the risk of overall macrovascular events and no adverse clinical consequences of developing signs of heart failure. Furthermore, there is good evidence of significant benefit regarding the composite of death, MI or stroke.

**Conclusion:**

The benefits seen with pioglitazone appear to outweigh the risks.

## INTRODUCTION — CONFUSION REIGNS IN THE WORLD OF TZDS

Clinicians have a wide range of different glucose-lowering drug options to call upon when selecting the most appropriate treatment for their patients with type 2 diabetes. In recent years, the two widely marketed thiazolidinediones (TZDs) — pioglitazone and rosiglitazone — have become a well-established component of treatment algorithms for the metabolic management of type 2 diabetes [1-3]. Both agents offer robust improvements in glycemic control that are at least comparable to those seen with established agents, such as metformin and the sulfonylureas [4-6]. More importantly, this effect appears to be remarkably durable with the TZDs, which may relate to their potential ß-cell preserving properties [7]. This is a particularly desirable property in a glucose-lowering agent, as it is the progressive failure and loss of ß-cells that is ultimately responsible for the onset and progression of type 2 diabetes [8]. When considering whether to use a TZD, clinicians also need to consider the potential impact of several other well-established characteristics of these agents, including their propensity to cause edema (and subsequently signs and/or symptoms of heart failure) and weight gain [9-13]. 

However, recent studies and analyses have added another characteristic of TZDs into the equation — their potential impact on cardiovascular (CV) events. Patients with type 2 diabetes are already at increased risk for heart failure and other adverse CV events and it would be cause for concern if this risk was increased further by glucose-lowering therapy. Several meta-analyses have suggested that there may be a signal for increased risk of myocardial ischemic events with rosiglitazone therapy, which has ignited debate and instilled uncertainty regarding the place of TZDs in diabetes treatment strategies [14,15]. Furthermore, these results appear to contrast with those for pioglitazone based on meta-analyses showing a reduction in CV events, as well as the results of the PROactive (PROspective pioglitAzone Clinical Trial In macroVascular Events) study, which provide the only robust CV outcomes data for a TZD to date [16,17]. 

The confusion is compounded further by uncertainty over the exact nature of the heart failure reported with both TZDs and whether it has any adverse consequences. Myocardial ischemic events are a consequence of reduced blood flow to the cardiac muscle. However, there is no evidence in the literature to suggest that the TZDs exert any direct adverse effect on cardiac function. Rather, the heart failure associated with TZD therapy may be associated with their established effects on fluid retention and, as such, may be manageable and/or reversible (for review see [18]). 

The debate sparked by the reports of increased myocardial ischemic events with rosiglitazone has led to a diverse range of individual ideas on the best course of action for patients on a TZD (especially rosiglitazone), including: no action; increased monitoring; switching to a non-TZD glucose-lowering drug; or switching from rosiglitazone to pioglitazone [19]. In this review, we consider the available evidence that allows an overall assessment of any potential (or genuine) CV clinical benefits or deficits of TZD therapy and ask whether the concern is justified for either rosiglitazone or pioglitazone. In particular, we consider both the class effects and drug-specific effects of the TZDs. 

## SHOULD WE EXPECT GLUCOSE-LOWERING DRUGS TO IMPACT ON CV OUTCOMES IN TYPE 2 DIABETES?

All current glucose-lowering agents, including the TZDs, were approved primarily on their ability to improve glycemic control with acceptable tolerability over relatively short-periods, a process that has at times received criticism (e.g. [20,21]). Unfortunately, this process does not allow the adequate assessment — prior to approval — of either long-term safety or benefit regarding relevant clinical outcomes, such as CV events. 

Whilst the need to reduce acute hyperglycemic crises and their serious consequences is readily apparent (and usually requires insulin therapy), it is the insidious long-term consequences of more moderate hyperglycemia and other metabolic disturbances that are most relevant to the patient with type 2 diabetes. Ultimately, it is the impact of diabetes on micro- and macrovascular disease that is responsible for much of the morbidity and mortality associated with the disease [22]).

Based on the limited clinical trial evidence available, it is assumed that reducing hyperglycemia over the long term, through whatever means, reduces the long-term risk of the microvascular and (probably) macrovascular complications associated with type 2 diabetes [23-26]. Unfortunately, the intensive blood glucose sub-study (target HbA_1c_ <6.0%) in the ACCORD (Action to Control CardiOvascular Risk in Diabetes) study of patients with type 2 diabetes and vascular disease/multiple CV risk factors has been stopped due to safety concerns (an increased mortality rate relative to the standard regimen, with an HbA_1c_ <7.0%), but data from the standard treatment arm will still provide invaluable insight into CV risk reduction. Most glucose-lowering drugs, including the TZDs, have multiple and differing effects on diverse biological processes (some of which may be potentially beneficial, others potentially detrimental) that may determine the effect on any particular clinical end point (e.g. cardiovascular disease [CVD]). Thus, while it is likely that lowering glucose is associated with reductions in vascular events, we cannot accurately predict the effect (good or bad) of any individual drug on CV (or any other) outcomes without suitable outcomes data. Unfortunately, adequate outcomes data are still lacking for the majority of glucose-lowering agents, in spite of some potential safety issues (Table **1**). 

## SHOULD WE EXPECT TZDS TO IMPACT ON CV OUTCOMES IN TYPE 2 DIABETES?

TZDs are agonists for nuclear peroxisome proliferator activated receptors (PPAR) and exert their glucose-lowering effect by binding to PPARγ. Metabolically, TZDs act as insulin sensitizers to improve glucose uptake, lower blood glucose and reduce hyperinsulinemia. Both pioglitazone and rosiglitazone have multiple effects on metabolic parameters that could potentially have an impact on CV outcomes (Table **2**), although there are some important differences between the two drugs. 

Certainly, both TZDs have been shown to produce clinically meaningful reductions in HbA_1c_, alongside low rates of hypoglycemia as either mono- or combination therapy and, therefore, provide a useful option when pursuing recommended glycemic goals [4,35]. Glycemic control with TZDs also appears to be particularly durable, as demonstrated in ADOPT (A Diabetes Outcome Prevention Study), where rosiglitazone was associated with significantly lower rates of monotherapy failure compared with either metformin or glyburide [7]. In addition to their ability to lower glucose, both drugs also have a small beneficial effect on blood pressure [36,37]. However, while both drugs modify the lipid profile in patients with type 2 diabetes, there are notable distinctions. As shown in a head-to-head comparison, rosiglitazone increases low-density lipoprotein cholesterol (LDL-C) concentration, increases the number of atherogenic (i.e. apo B100-containing) particles and tends to raise triglycerides, whereas pioglitazone is neutral with respect to LDL-C levels (but does change favorably the size and concentration of LDL particles), tends to lower apo B100 and reduces plasma triglyceride levels [38,39]. Additionally, pioglitazone is more effective at raising high-density lipoprotein cholesterol (HDL-C) and converting small, dense LDL particles to larger, more buoyant ones [38,39]. Accordingly, significant improvements in triglycerides, total cholesterol and HDL-C have been reported when patients are switched from rosiglitazone to pioglitazone, while glycemic control remains stable [40]. 

In addition to their impact on well-established risk factors, both TZDs also have potentially beneficial effects on a myriad of non-traditional risk markers associated with vascular function and CVD (for reviews, see [41-43]). Among these, TZDs improve markers of inflammation (e.g. C-reactive protein [CRP]), influence components of the coagulation cascade (e.g. plasminogen activator inhibitor-1 [PAI-1]) and increase levels of the anti-atherosclerotic adipokine, adiponectin [44-47]. They also modulate processes involved in macrophage foam cell formation, plaque stability and the response to vascular injury, as well as improving endothelial function and microalbuminuria [43,48]. Studies in animal models also demonstrate their ability to improve outcomes after experimentally induced myocardial infarction (MI) or stroke [49-52]. In human studies, they also improve cardiac performance and pioglitazone has been shown to reduce the progression of carotid intima-media thickness (CIMT), which is a well-established surrogate for atherosclerosis [53,54]. Most recently, pioglitazone was shown to reduce the progression of atherosclerosis, as measured using intravascular ultrasound, and improve CV risk factors over 18 months, whereas there was a progression of coronary atherosclerosis with glimepiride [55].

**Fig. (1). Different thiazolidinediones have only partially overlapping gene expression profiles:**
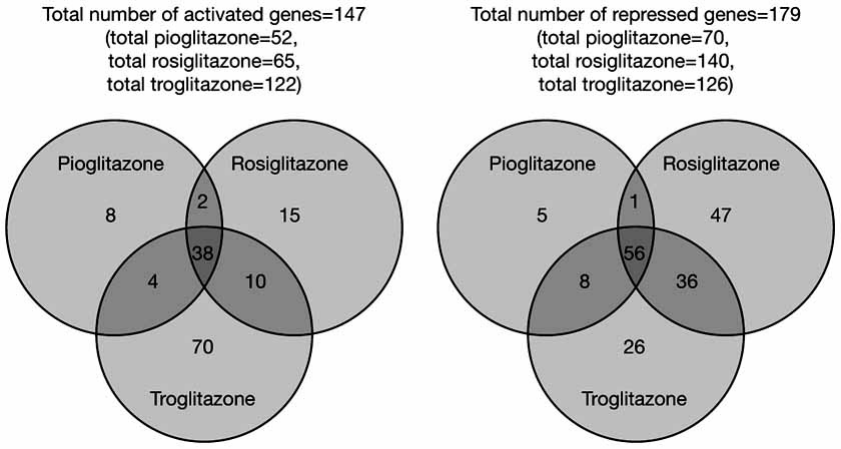
The total number of genes regulated by a particular TZD is shown next to its name. The number of genes uniquely regulated by a particular TZD is contained in the non-overlapping regions of each circle. The numbers of genes similarly regulated by two or three TZDs are contained in the overlapping regions of the circles [57]. (Reprinted from Biochem Biophys Res Commun, 364(3), Sears DD, Hsiao A, Ofrecio JM, Chapman J, He W, Olefsky JM, Selective modulation of promoter recruitment and transcriptional activity of PPARgamma, 515-521, Copyright © 2007, with permission from Elsevier).

Differential gene expression between TZDs, based on the selective PPAR modulator (SPPARM) model of PPARγ ligand action, may provide a mechanistic explanation for some of the differences within this class of drugs [56,57]. According to this model, the ligand-receptor (i.e. TZD-PPARγ) complex for each TZD takes on a different conformation, resulting in distinct patterns of interactions with nuclear cofactors, histones and other transcription factors, etc. and, consequently, leading to different patterns of gene expression for each individual TZD [57,58]. For instance, a recent study showed complex, only partially-overlapping gene expression profiles for over 300 genes regulated by troglitazone, rosiglitazone and pioglitazone (Fig. **1**) and among the common genes, time course and, dose-response studies also revealed further differentiation in terms of TZD-specific expression kinetics [57]. Furthermore, recruitment among the myriad of nuclear receptor coactivators, co-repressors and coregulators — protein moieties that appear to play a critical role in transcriptional regulation influencing a wide variety of biological processes — presents a whole new level of complexity that may depend on their tissue specificity, and their interactions with each other and with other signalling pathways [58-61]. As such, TZD-specific interactions with these nuclear cofactors may be relevant to differential effects of individual TZDs in different tissues and under different metabolic conditions. Several PPARγ-independent off-target effects that may contribute to the CV risk-benefit profile of individual TZDs have also been reported [62,63].

Thus, based on their multiple effects on glycemia, lipid profiles, blood pressure, biomarkers and surrogate CV endpoints, we would anticipate that TZDs have the potential to influence CV outcomes positively. At the same time, it would not be surprising if individual TZDs differed in the nature and/or extent of that impact, given their differing effects on gene expression and lipids. As noted above, rosiglitazone (but not pioglitazone) may have some potentially detrimental absolute effects on lipids (notably, increased LDL-C concentration and apoB100-containing particle number), and any potentially beneficial effects of rosiglitazone on lipids (e.g., increased HDL-C concentration and increased LDL particle size) appear to be more marked with pioglitazone, which also has the additional benefits of lowering triglycerides and apoB100-containing particle number. The greater effect of pioglitazone on HDL-C may be particularly relevant, as it appears to be a key factor underlying the significant slowing of CIMT progression seen with this TZD in patients with type 2 diabetes [64].

## WHAT ARE THE OBSERVED EFFECTS OF TZDS ON ACTUAL CLINICAL OUTCOMES?

### Macrovascular Events

#### Pioglitazone

**Fig. (2). Pioglitazone decreases the risk of major macrovascular events:**
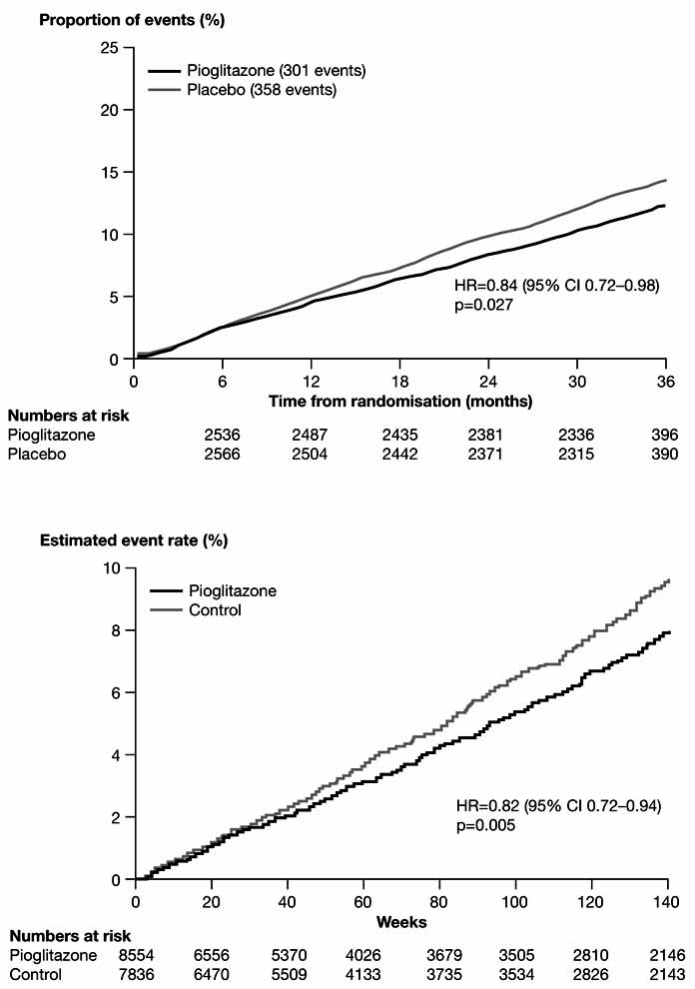
Data are for the composite of all-cause mortality, MI and stroke from (A) PROactive and (Reprinted from The Lancet, 366, Dormandy JA, Charbonnel B, Eckland DJ; PROactive investigators, Secondary prevention of macrovascular events in patients with type 2 diabetes in the PROactive Study (PROspective pioglitAzone Clinical Trial In macroVascular Events): a randomised controlled trial, 1279–1289, Copyright © 2005, with permission from Elsevier). (B) a meta-analysis of 19 randomized controlled trials [16,17]. (Reprinted with permission from Lincoff AM et al, JAMA 2007; 298: 1180–1188, Copyright © 2007 American Medical Association. All rights reserved).

The most robust data on CV outcomes with TZDs comes from the PROactive trial, which involved over 5000 patients with type 2 diabetes and established macrovascular disease [16,65-69]. This prospective, randomized, placebo-controlled study showed that, over approximately 3 years, pioglitazone therapy was associated with a statistical trend towards benefit (hazard ratio [HR]=0.90, 95% confidence interval [CI] [0.80, 1.02], p=0.095) for the primary composite macrovascular end point (a complex composite of cerebral, cardiac and peripheral events and both disease-related and procedural end points). A statistically significant reduction in the main secondary endpoint of the composite of all-cause mortality, MI or stroke (HR=0.84, 95% CI [0.72, 0.98], p=0.027) was also reported (Fig. **2**), along with significant effects on several other major adverse cardiovascular events (MACE) end points [16,68]. 

These results are supported by a recent meta-analysis of 19 randomized controlled trials (RCTs) involving pioglitazone, which showed a significant decrease in the composite of death, MI or stroke (HR=0.82, 95% CI [0.72, 0.94], p=0.005) and a non-significant decrease in the risk of MI (HR=0.81, 95% CI [0.64, 1.02], p=0.08) relative to comparator therapies (Fig. **2**), and these results held when PROactive was omitted from the analyses [17]. Also notable in PROactive were statistically significant reductions in the risk of recurrent MI (HR=0.72, 95% CI [0.52, 0.99], p=0.045) or recurrent stroke (HR=0.53, 95% CI [0.34, 0.85], p=0.009) [65,66]. In addition, pioglitazone also appeared to be particularly effective at reducing macrovascular events in patients with chronic kidney disease, but was relatively ineffective in patients with peripheral arterial disease (PAD) at baseline [67,69]. There was, however, an excess of leg revascularizations in patients treated with pioglitazone — this was restricted to those patients with evidence of PAD at baseline (N=1274), and most of the excess events occurred in the first year of the study when a total of 42 leg revascularization events occurred in the pioglitazone arm versus 24 events in the placebo arm, compared with 39 and 41 events, respectively, during the second and third years of the study [69].

#### Rosiglitazone

**Fig. (3). Available safety data for rosiglitazone and pioglitazone in terms of macrovascular risk relative to comparators:**
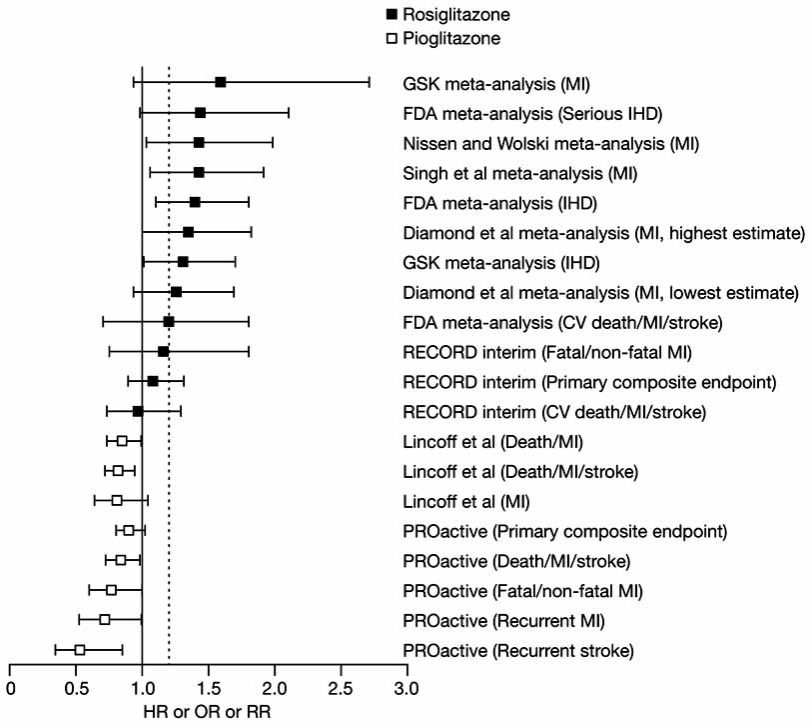
Data are from meta-analyses, the interim results of the RECORD trial and the PROactive trial. The primary endpoint in RECORD was the composite of hospitalization or death due to CV causes. The primary endpoint in PROactive was the composite of all-cause mortality, MI (incl. silent MI), stroke, ACS, coronary revascularization, major leg amputation and leg revascularization. The dotted line represents the non-inferiority limit (1.2) for the upper CI in the RECORD study FDA=(US) Food and Drug Administration; GSK=GlaxoSmithKine; IHD=ischemic heart disease; OR=odds ratio; HR=hazard ratio; RR=relative risk. (No permission required).

In contrast to the findings with pioglitazone, several separate meta-analyses of RCTs involving rosiglitazone have raised the possibility of an increased risk of MI and/or ischemic cardiac events versus comparators, although it should be emphasised that none of the included studies were designed specifically to assess CV events and the studies have used different measures for relative risk (some have used HR, some have used the odds ratio [OR] and some relative risk [RR]) (Fig. **3**) [14,15,70,71]. While the methods used to perform these meta-analyses have been the subject of considerable debate and consequently re-analysed using different criteria (e.g. [72]), they all showed a trend towards increased risk. 

Using CV adverse event summary data from 42 studies (N=27,847) identified in the literature and other sources, Nissen and Wolski found that rosiglitazone was associated with a significant 43% increase in the risk of MI versus all comparators (OR=1.43, 95% CI [1.03, 1.98], p=0.03) and a 64% non-significant increase in CV death (OR=1.64, 95% CI [0.98, 2.74], p=0.06) [14]. However, this apparent increased risk of CV death has not been reported in other meta-analyses of rosiglitazone outcomes and, when these data were analysed using various different (arguably more appropriate) statistical corrections, the increase in MI did not achieve statistical significance [72]. Two separate independent unpublished meta-analyses (performed by GlaxoSmithKline and the US Food and Drug Administration [FDA]) of patient-level data from a different dataset of 42 studies both showed a significant increase in myocardial ischemic events with rosiglitazone (Fig. **3**) [15,70]. A subsequent analysis of MI alone from this dataset, however, showed an increased risk that failed to reach significance (OR=1.59, 95% CI [0.93, 2.71]) [73]. A significant increase in MI with rosiglitazone was also reported in another separate meta-analysis that was restricted to the four long-term (≥12 months) studies only (RR=1.42, 95% CI [1.06, 1.91], p=0.02) [55]. Despite this, an increase in the incidence of MACE end points has not been consistently reported in meta-analyses of rosiglitazone outcomes. 

These meta-analyses currently represent the best CV safety data available for rosiglitazone. Interim results from the ongoing RECORD (Rosiglitazone Evaluated for Cardiac Outcomes and Regulation of Glycaemia in Diabetes) trial — a prospective, randomized, open-label, uncontrolled non-inferiority CV outcomes study with rosiglitazone — have been inconclusive, and it is difficult to make any conclusions from this early underpowered analysis [74]. RECORD was designed as a non-inferiority study comparing rosiglitazone (plus metformin or a sulfonylurea) with metformin and a sulfonylurea. Success was defined as the upper bound of the 95% CI being less than 1.2 (a >20% increased risk in the primary end point of a composite of hospitalization or death from CV causes, including heart failure) with rosiglitazone. After 3.75 years, the risk of reaching the primary end point in the rosiglitazone group was not significantly different from the comparator group (HR=1.08, 95% CI [0.89, 1.31, p=0.43). However, the upper CI exceeded the predefined non-inferiority limit of 1.2 (Fig. **3**). Similarly, there was no significant difference with regards to the important secondary composite end point of CV death, MI and stroke or the secondary end point of MI alone (HR=1.16, 95% CI [0.75, 1.81]), p=0.50 (Fig. **3**). The risk of MI alone increased when events pending adjudication were included in the analysis (HR= 1.23, 95% CI [0.81, 1.86], p=0.34). It remains to be seen whether RECORD will ever have enough power to provide an authoritative answer regarding the ischemic myocardial danger signal noted in the rosiglitazone meta-analyses, as it has had a much lower than expected event rate and a much higher than expected drop-out rate. 

### Observational and Cohort Data

Observational studies represent another source of data that can be considered alongside RCTs and meta-analyses, but they should only be used for signal detection as they can only demonstrate an association and are subject to uncontrollable confounding and bias. A negative observational study does not mean a signal does not exist, merely that it has not been seen. In a recent retrospective cohort study from Canada, current TZD monotherapy was associated with a significantly increased risk of hospitalization/admission for MI compared with other oral agent therapy [75]. When the data for individual TZDs were examined, the significant association persisted for rosiglitazone (RR=1.76, 95% CI [1.27, 2.44], p<0.001). Although there was no comparable increase in risk with pioglitazone, the analysis was limited by the smaller number of patients receiving that agent. Another study found a significant difference in the risk of MI and coronary revascularization between sulfonylureas (higher risk) and metformin (lower) risk, but no significant difference when either agent was compared with rosiglitazone, which appeared to impart a level of risk somewhere between the two [76]. A retrospective cohort study comparing rosiglitazone and pioglitazone appears to support the results of the meta-analyses [77]. The risk of hospitalization for MI was significantly lower for pioglitazone compared with rosiglitazone (HR=0.78, 95% CI [0.63, 0.96]), as was the risk of the composite of MI and coronary revascularization (HR=0.85, 95% CI [0.75, 0.98]). However, other observational studies have found no increased risk with rosiglitazone relative to other oral glucose-lowering agents (the OR for the risk of MI, CV death or stroke was found to be 1.2, p=040 for rosiglitazone versus comparators in the Rosen FDA analysis of 42 trials) [15].

### Edema, Weight Gain and Signs of Heart Failure

Data from several sources (PROactive and RECORD, non-CV outcome RCTs, meta-analyses and observational studies) have established that both rosiglitazone and pioglitazone are associated with edema, weight gain and signs of heart failure [10-13,18,78]. Weight gain with pioglitazone appears to be associated primarily with fluid retention and there is currently no available evidence to suggest that this sort of weight gain is associated with any adverse macrovascular outcomes [79]. Importantly, however, heart failure that develops while on TZD therapy appears to be the result of sodium-water retention rather than any adverse effect on the myocardium (for review see [18]). In fact, some evidence suggests that TZDs may actually improve cardiac function (for review see [18]). These characteristics of TZD-induced heart failure were evident in analyses from PROactive. Although the risk of serious heart failure was clearly greater with pioglitazone, absolute macrovascular event rates were similar to placebo [79]. In fact, a time-to-event analysis among those patients developing signs of serious heart failure while on pioglitazone suggested a proportional decrease in event rates and mortality compared with those on placebo. The potential impact of heart failure associated with rosiglitazone therapy on CV outcomes has not been assessed.

## HOW CAN WE DECIDE WHICH PATIENTS WILL BENEFIT MOST (OR LEAST) FROM TZD THERAPY?

Unfortunately, there is relatively little robust evidence available to help to predict which patients might be at increased cardiac ischemic risk with rosiglitazone. Data from the various meta-analyses suggest that the risk may be higher in those on nitrates or concomitant insulin therapy or those with previous established CVD [15,70]. The US prescribing information for rosiglitazone recommends against combination with nitrates or insulin. 

There is good evidence from PROactive to suggest which patients might benefit most or least from secondary prevention therapy with pioglitazone. Firstly, patients with a history of MI or stroke derived particularly marked reductions in the risk of recurrent events [65,66]. Secondly, the presence of renal dysfunction does not appear to be a limiting factor and patients with chronic kidney disease may in fact derive greater macrovascular benefit [67]. Patients with evidence of pre-existing occlusive PAD, however, would appear not to be good candidates, because pioglitazone seems to be relatively ineffective at reducing macrovascular events in these patients and also because pioglitazone may be associated with an increase in leg revascularizations in this patient group [69]. Finally, it should be emphasised that PROactive cannot provide any insights into the impact of pioglitazone on primary CV events, possibly because the exposure to treatment was too short.

Both TZDs carry warnings regarding their propensity to cause edema and heart failure and the need for appropriate management of these symptoms. In the European Union, TZDs are contraindicated in patients with cardiac failure or history of cardiac failure (New York Heart Association [NYHA] Stages I to IV), whereas in the US their use is only contraindicated in those with Stage III-IV heart failure. For both TZDs, a key factor is predicting those patients who are most likely to develop edema and signs of heart failure (although, as noted above, there may be no adverse consequences associated with this, at least for pioglitazone). Rapid weight gain may be a sign of fluid retention and could indicate the potential to develop edema and signs of heart failure. Furthermore, diagnostic techniques, such as brain natriuretic peptide measurement, may help to identify those patients likely to develop heart failure with TZD treatment and could help to establish whether symptoms reflect heart failure or simply volume overload [18]. Edema from causes not related to heart failure should not preclude TZD use and may be readily amenable to diuretic therapy [18].

**Fig. (4):**
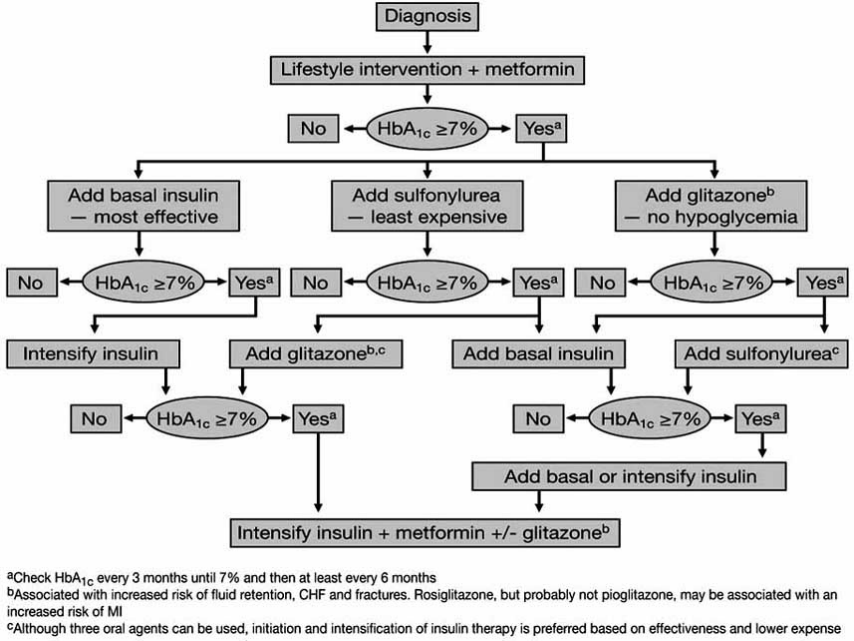
Algorithm for the metabolic management of type 2 diabetes mellitus [1,2]. (Reprinted with permission from Nathan DM, et al, Management of hyperglycaemia in type 2 diabetes: a consensus algorithm for the initiation and adjustment of therapy. A consensus statement from the American Diabetes Association and the European Association for the Study of Diabetes, Diabetologia 2006; 49: 1711–1721, Copyright © Springer-Verlag 2006).

Both the European Medicines Evaluation Agency (EMEA) and the FDA have concluded that the benefits of both TZDs continue to outweigh any possible detrimental effects [15,80]. However, in the US, the FDA has recently added a black box warning for myocardial ischemia to the rosiglitazone labelling. In Europe, the EMEA has added the contraindication of an acute coronary syndrome (unstable angina, ST segment elevation MI [STEMI] and non-STEMI) on the labelling for rosiglitazone-containing products [81]. No similar changes or recommendations have been made for pioglitazone and pioglitazone is indicated for use in patients who are receiving insulin. The recently updated American Diabetes Association (ADA)/European Association for the Study of Diabetes (EASD) consensus algorithm also highlights the potential of rosiglitazone to increase MI and of pioglitazone to decrease MI (Fig. **4**) [2].

## CONCLUSION — SHOULD CONFUSION REIGN IN THE WORLD OF TZDS?

The TZDs are drugs with complex mechanisms of action that have multiple biological effects with the potential to influence various clinical outcomes. Gene expression studies have highlighted the marked differential effects of individual TZDs on a whole range of genes, although the functional consequences of these differences and their relevance in terms of CV risk remain to be elucidated. Furthermore, clinical studies suggest that the metabolic profiles of individual TZDs only partially overlap, particularly with regards to important lipid parameters, such as HDL-C. Thus, there is a sound justification (albeit hypothetical) for predicting that individual TZDs might have differing effects on CV outcomes, despite their similar effects on glycemic control.

For pioglitazone, at least, we can be relatively certain that there is no net increase in the risk of overall macrovascular events (based on both PROactive and meta-analyses) and no adverse clinical consequences of developing signs of heart failure (based on PROactive). The main secondary outcome of PROactive and a meta-analysis of RCTs provide good evidence of a significant benefit regarding the composite of death, MI or stroke. These outcomes data are important when assessing the overall clinical profile of pioglitazone.

Similar robust data for rosiglitazone are lacking. The signal for increased ischemic cardiac risk reported for rosiglitazone based on data from meta-analyses has brought several key issues to the forefront. Firstly, it has led clinicians to question whether there are sufficient data available to suggest that rosiglitazone (or many other diabetes agents) provides a net beneficial effect on important clinical outcomes. Secondly, the available studies suggest that any evidence of clinical benefit (or harm) with one TZD cannot be extrapolated to another, and there are plausible mechanistic reasons for important differences. Thirdly, it has re-emphasised that the ability of a drug to lower glucose may not be sufficient in itself to have a beneficial effect on macrovascular outcomes [82]. 

We conclude that the benefits with both TZDs continue to outweigh the risks. Based on currently available data, there is good evidence to suggest that pioglitazone is not associated with an increased risk of ischemic CV events and may in fact provide an overall CV benefit (and an overall clinical benefit). Although there are reports from meta-analyses that there is a risk of MI with rosiglitazone, there are no outcomes data to support this suggestion. Thus, we believe that, for pioglitazone at least, there is no reason for patients or physicians to be alarmed when making judicious use of this agent in appropriate patients. It has been suggested very recently that pioglitazone represents a reasonable next option in patients with type 2 diabetes who have macrovascular disease (not complicated by heart failure or not limited to the leg) in whom adequate glycemic control is not being achieved with metformin alone [27]. 

## Figures and Tables

**Table 1:** Known or Potential Cardiovascular Issues with the Major Classes of Oral Glucose-Lowering Agents

Oral Agent Class	Known or Potential Cardiovascular Issues
Sulfonylureas (Glyburide, Glipizide, Glimepiride, Others)	Hypoglycemia may precipitate ischemia, arrhythmia [27] Cardiac K_ATP_ channel closure may impair ischemic preconditioning (this may be more important with specific agents, notably glyburide) [27,28] Long-term outcomes trials suggest no harmful CV effects when used as part of an intensive glucose control strategy (based on the UKPDS and ADVANCE) [23,29] Potential harmful effect on outcomes when used in combination with metformin (based on the UKPDS and a meta-analysis of observational studies) [24,30]
Glinides (Nateglinide, Repaglinide)	Hypoglycemia may precipitate ischemia, arrhythmia [27] Cardiac K_ATP_ channel closure may impair ischemic preconditioning (this may be more important with repaglinide) [27,31] No long-term data regarding CV safety and efficacy (data for nateglinide in prediabetes will be available soon from NAVIGATOR) [32]
Biguanides (Metformin)	May improve CVD outcomes in overweight patients when used as the basis of an intensive glucose control strategy (based on a relatively small study embedded in the UKPDS) [24] Should not be used in acute or unstable HF because of lactic acidosis risk [27,33] Potential harmful effect on outcomes when used in combination with sulfonylureas (based on the UKPDS and a meta-analysis of observational studies) [24,30]
α-glucosidase inhibitors (Acarbose, Miglitol)	Improves postprandial glucose excursions, which are more tightly associated with CVD than fasting glucose [27,34] May reduce MI risk in prediabetes (based on a small number of events in STOP-NIDDM) or type 2 diabetes (meta-analysis of RCTs) [27,34]
Thiazolidinediones (Rosiglitazone, Pioglitazone)	May precipitate clinical HF in predisposed individuals [27] Pioglitazone may reduce MI, stroke risk (based on PROactive and meta-analyses of RCTs) [27] Rosiglitazone may increase MI risk (based on meta-analyses of RCTs) [27]
DPP-4 inhibitors (Sitagliptin, Vildagliptin)	No long-term data regarding CV safety and efficacy [27] Effects on CV risk entirely unknown [27]

Table adapted from ref. [27] ADVANCE=Action in Diabetes and Vascular Disease: Preterax and Diamicron Modified Release Controlled Evaluation STOP-NIDDM=Study TO Prevent Non-Insulin-Dependent Diabetes Mellitus

**Table 2:** Key Factors to Consider when Determining the Net Clinical Benefit of either Pioglitazone or Rosiglitazone

**Pioglitazone**	

***Beneficial/Potentially Beneficial Factors***	***Detrimental/Potentially Detrimental Factors***

Reduced risk of all-cause mortality, MI and stroke (based on PROactive and meta-analyses) and no evidence of increased risk Reduced risk of recurrent MI Reduced risk of recurrent stroke Reduction in restenosis/repeat TVR (relevant only in patients undergoing PCI) Effects on traditional metabolic risk factors (glucose, triglycerides, HDL-C, blood pressure, LDL particle concentration) Effects on surrogate endpoints (CIMT, IVUS) Effects on non-traditional risk markers	Increase in edema and weight gain Increase in signs of heart failure (which is not associated with adverse CV outcomes) Possible increase in peripheral revascularization in patients with evidence of occlusive PAD Possible increase in distal fractures (postmenopausal women)

**Rosiglitazone**	

***Beneficial/Potentially Beneficial Factors***	***Detrimental/Potentially Detrimental Factors***

Reduction in restenosis/repeat TVR (relevant only in patients undergoing PCI) Effects on traditional metabolic risk factors (glucose, HDL-C, blood pressure) Effects on surrogate endpoints (CIMT) Effects on non-traditional risk markers	Signal for increased cardiac ischemic events (based on meta-analyses) Increase in edema and weight gain Increase in signs of heart failure Possible increase in distal fractures (postmenopausal women) Metabolic effects (increased LDL concentration and particle number)

CIMT=carotid intima-media thickness; PCI=percutaneous coronary intervention; IVUS=intravascular sonography; TVR=target vessel revascularization
